# Headache

**Published:** 1885-06

**Authors:** 


### EDITORIAL, ETC.

Headache.—The following comments are made by the *Medical and Surgical Reporter*, quoting an article from the London *Lancet* on "Headache."

"It is such a common thing to hear a person say, "*I have a headache*," and it is as equally common to hear the reply, "*It will soon pass off*," and we so constantly find such recommendations for its relief as a cup of tea, or a nap, or "vinegar and brown paper," and we so seldom inquire into the *cause*, unless the headache is so continuously persistent or some other prominent symptoms imperiously force our attention to a close examination, that we reproduce the following remarks from the *Lancet* as well worthy of careful consideration:"

"Headache, although so common an affection, possesses a peculiar interest on account of the tantalizing obscurity in which its cause is often shadowed. Depending upon one or two main physiological principles connected with the passage of nervous impulses and the calibre of cerebral blood-vessels, it yet arises in response to so many different stimuli, that, practically, the branches of its original stem of structure, its distributive variations, are perplexingly numerous. Among the forms but recently described and named, though probably as old as art, is the 'academy headache.' Critics and doctors have vied with one another in trying to translate this term according to the rules of medical logic, but have not, so far, been able to agree as to its meaning. In venturing to put forward our own opinion on the subject, we think it best to notice the different circumstances which tend to produce in the frequenter of galleries that general sense of fatigue which is often the precursor of pain, and then to note the special stimulus which brings out of this chaos the formed and local ache. It should be noted that all do not suffer from this form of headache· while of those who do, some are less and some are more affected. Fatigue may be the limit of annoyance with a certain class. They are heavy-limbed and heavy-headed, languid, but no more. The forces at work upon them are the warmth of the crowded room, the summer sunshine pouring through the skylights, the listless rotation or circulation, rather than walking, through the rooms, and the weariness of much divided attention. Being robust and used to go about upon their feet, they soon lose the feeling of languor after its causes cease to act; and if their eyesight be equally healthy, they suffer no strain in these organs. There are other persons, of weaker organization or more sedentary habits, in whom pain quickly follows fatigue, and it is felt, for certain reasons, chiefly in the head. Among these reasons we must note the fact that in the standing position the blood, driven by a languid heart, reaches the head with greater difficulty than when recumbent. Anæmia, is one cause of headache. Anæmia, too, is associated with changes of arterial calibre which constitute the usual cause of megrim. Under these conditions, also, the giddy malaise of a loitering gait is painfully perceptible. Concentrated attention increases the uneasy sensation. The frequently constrained position of the head has its influence. It would be strange, moreover, if ocular movements had no share in the production of this complex indisposition. Let any one converge his eyeballs to an acute angle, as in squinting, or let him direct a sidelong glance of extreme obliquity, and a single effort will convince him of the pain of such muscular effort. No doubt this is the pain of muscular tension, acute and so far abnormal; but it is also possible to induce a similar pain from the strain of too frequent muscular action within the normal limits of accommodation to distance or other movements of the eye. Ocular pains of this kind are felt as an aching about the middle and back of the head. The scrutiny of paintings or engravings, from the many points of interest which have to be noted, requires a considerably greater exercise of the muscles of the eyeball than is called for in ordinary life. In this fact, established as we have already stated, on a basis of fatigue and general muscular and nervous strain, we have probably the usual exciting cause of the form of headache we have been discussing.

